# Echocardiography in the cardiac assessment of young athletes: a 2025 guideline from the British Society of Echocardiography (endorsed by Cardiac Risk in the Young)

**DOI:** 10.1186/s44156-025-00069-0

**Published:** 2025-03-14

**Authors:** David Oxborough, Keith George, Robert Cooper, Raghav Bhatia, Tristan Ramcharan, Abbas Zaidi, Sabiha Gati, Keerthi Prakash, Dhrubo Rakhit, Shaun Robinson, Graham Stuart, Jan Forster, Melanie Ackrill, Daniel Augustine, Aneil Malhotra, Michael Papadakis, Silvia Castelletti, Victoria Pettemerides, Liam Ring, Antoinette Kenny, Aaron Baggish, Sanjay Sharma

**Affiliations:** 1 Research Institute for Sport and Exercise Sciences and the Liverpool Centre for Cardiovascular Science at Liverpool John Moores University, Tom Reilly Building, Byrom Street, Liverpool, L3 3AF UK; 2 Hull University Teaching Hospitals NHS Trust, Kingston‑Upon‑Hull, UK; 3 Heart Unit, Birmingham Women’s and Children’s NHS Foundation Trust, Birmingham, UK; 4 University Hospital of Wales, Cardiff, UK; 5 Department of Cardiology, Royal Brompton & Harefield NHS Foundation Trust, London, UK; 6 Bradford Teaching Hospitals NHS Foundation Trust, Bradford, UK; 7 University Hospital Southampton NHS Foundation Trust, Southampton, UK; 8 Imperial College Healthcare NHS Trust, London, UK; 9 Bristol Heart Institute, Bristol, UK; 10 Leeds Teaching Hospitals NHS Trust, Leeds, UK; 11 University Hospital Dorset, Dorset, UK; 12 Royal United Hospitals Bath, Bath, UK; 13 Institute of Sport, Manchester Metropolitan University, Manchester, UK; 14 Cardiovascular Clinical Academic Group and Cardiology Research Centre, St. George’s, University of London, London, UK; 15 Cardiology Department, IRCCS Istituto Auxologico Italiano, 20149 Milan, Italy; 16 Liverpool Heart and Chest Hospital, Liverpool, UK; 17 West Suffolk Hospital NHS Trust, Bury St Edmonds, UK; 18 Cardiothoracic Centre, Freeman Hospital, Newcastle, UK; 19 Institut Des Sciences du Sport, Universite de Lausanne, Lausanne, Switzerland

**Keywords:** Athlete, Echocardiography, Guideline, Sport

## Abstract

Sudden cardiac death in a young physically active individual or athlete is a rare but tragic event. Pre-participation screening and follow-up investigations are utilised to reduce the risk and occurrence of these events. Echocardiography plays a key role in the cardiac diagnostic pathway and aims to identify underlying inherited or congenital structural cardiac conditions. In 2013 the British Society of Echocardiography and Cardiac Risk in the Young produced a joint guidance document to support echocardiographers in this setting. The document was subsequently updated in 2018, and it is now timely to provide a further update to the guideline drawing on the advances in our knowledge alongside the developments in ultrasound technology within this nuanced area of sports cardiology.

**Graphical Abstract:**

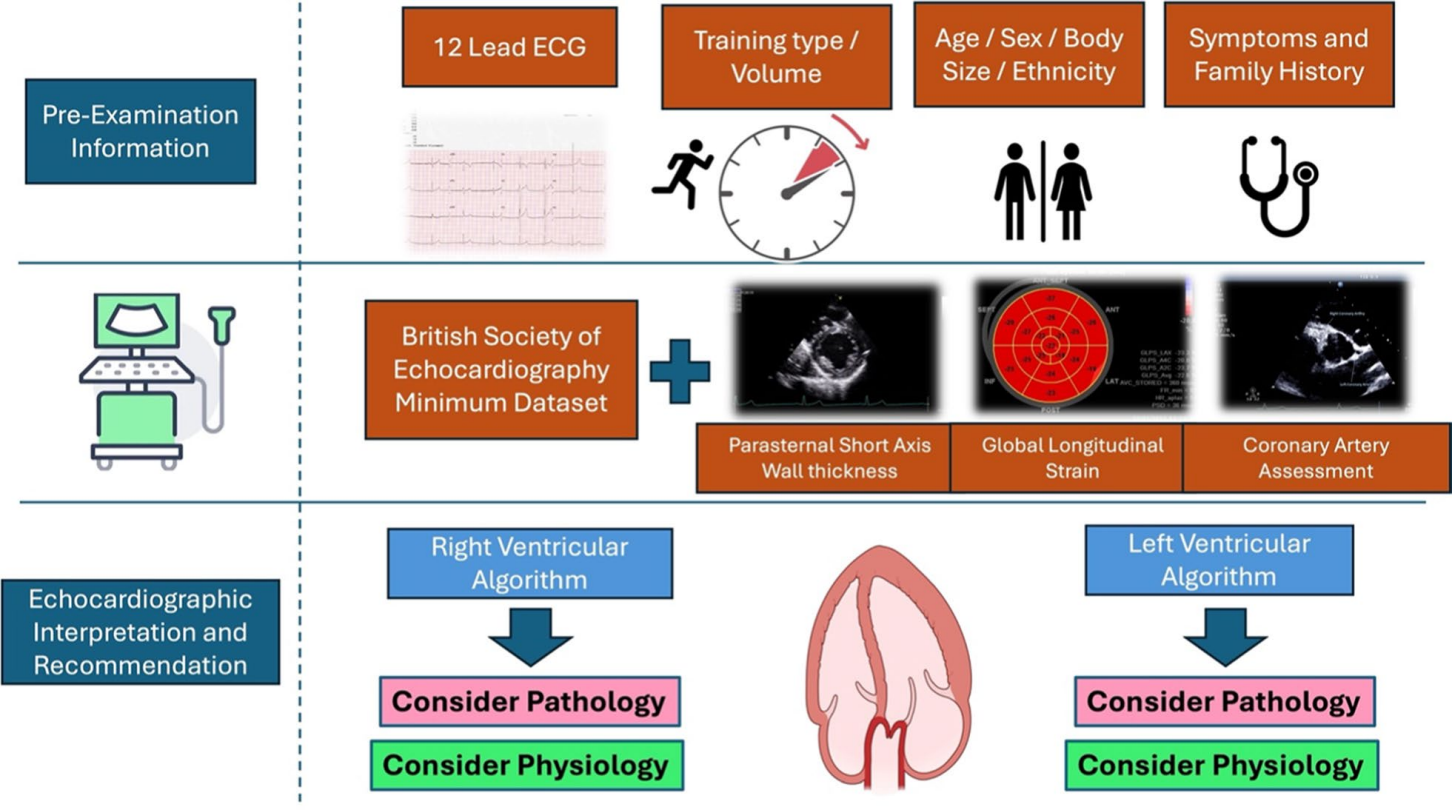

## Introduction

Echocardiography plays an important role in the cardiac assessment of young (typically 14–35 years old) individuals who engage in greater than 3 h of structured exercise per week [1] (termed *athlete* from here onwards). The aim of echocardiography in the athlete, is to identify or exclude structural and functional abnormalities consistent with underlying cardiac disease which may predispose them to an acute cardiac event or sudden cardiac death (SCD). This can be achieved in isolation and/or in conjunction with other diagnostic investigations and hence in 2013 the British Society of Echocardiography (BSE) (supported and endorsed by Cardiac Risk in the Young) published guidance on the standardised echocardiographic approach to image acquisition and interpretation in sports participants which was subsequently updated in 2018. Since the 2018 update, our understanding in this field has advanced considerably and there have been significant refinements in echocardiographic techniques to distinguish physiological cardiac adaptation from structural diseases implicated in SCD in the young athletic population. It is therefore timely to update the guidelines to reflect these advancements. The aim of this iteration is to provide a working document that can be used to support healthcare practitioners for a comprehensive and systematic approach to acquisition and interpretation of the echocardiogram in athletes including guidance related to the added value of exercise stress echocardiography (ESE), strain imaging and 3-dimensional echocardiography (3DE) in this important field of sports cardiology.

## Background

### Sudden cardiac death in young athletes

The death of a young athletic person is devastating with the premature loss of life alongside the significant impact on their family and the wider community. These tragic events are even more difficult to comprehend when they occur in individuals who are considered healthy and have engaged in regular sporting activities. The prevalence and incidence of SCD in young athletes is not fully elucidated due to challenges associated with compiling data from often unreliable and incomplete sources. This is compounded by a lack of structured registries and the absence of longitudinal data which may, in part, explain the large range of the reported prevalence of SCD in athletes (1 in 14,000 to 1 in 100,000) [2, 3]. Data also highlight that athletes have a 6–8 fold increased risk of SCD compared to non-athletes of the same age with male athletes and those of black ethnicity being at the greatest risk [4, 5]. Many of these adverse events occur during exercise [4] in athletes who are already predisposed and harbouring pre-existing cardiac disease. This observation is the fundamental rationale for pre-participation cardiac screening (PPCS) to identify vulnerable athletes.

Structural cardiac conditions responsible for SCD in athletes are primarily inherited but can also be congenital and acquired. Recent data from a large UK database of 748 decedents [4] who had engaged in regular sporting activities demonstrated that although there was a predominance of sudden arrhythmic death syndrome (SADS), 42% of males and 30% of females had conditions that could have been detectable on echocardiography. These conditions include hypertrophic (HCM), dilated (DCM) and arrhythmogenic cardiomyopathy (ACM), coronary artery anomalies (CAA), idiopathic left ventricular (LV) fibrosis, aortic dissection, valve disease and myocarditis [5–7]. The recent global SARS-Cov-2 health pandemic has also provided concern regarding the impact of viral myocarditis in the athletic / active population. Data from North America and Europe suggest that cardiac abnormalities following COVID-19 infection in these populations ranges from 0.2% to 4% [8, 9] and the established associations between myocarditis and SCD [10, 11] mean that consideration of cardiac evaluation in symptomatic athletes (including echocardiography) may help to reduce any additional risk.

### The role of echocardiography

**Fig. 1:**
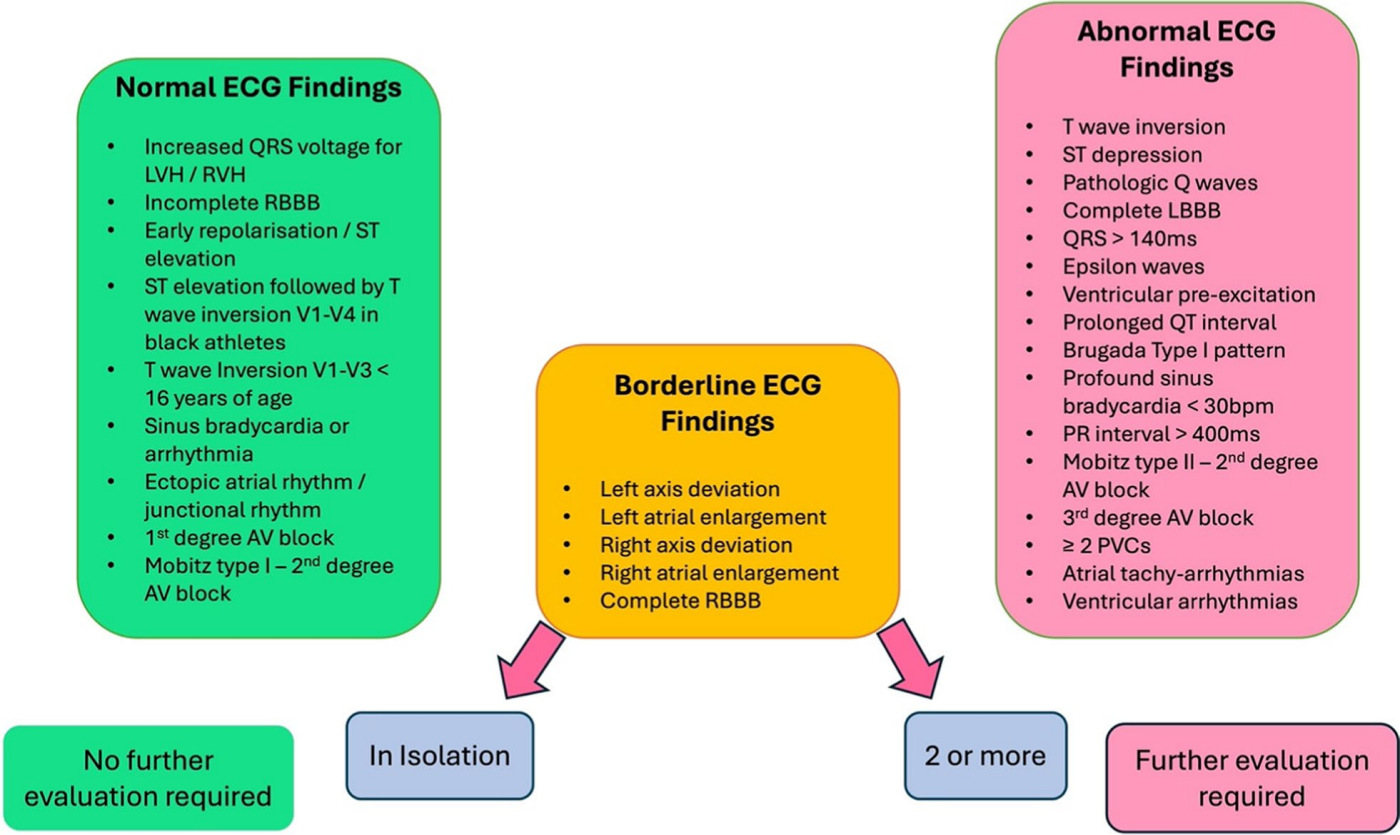
12-lead ECG criteria for onward referral to Echocardiography as per the International Guidelines for Electrocardiographic Interpretation in Athletes (adapted from Sharma et al. 2018)

Pre-participation cardiac screening has been shown to effectively diagnose underlying cardiac conditions and reduce the risk of SCD in young athletes between 14 and 35 years old [2, 12–14]. Echocardiography provides a non-invasive, easily accessible and relatively inexpensive means of accurately assessing cardiac structure and function in athletes, leading to its integration into various PPCS and follow-up protocols [15]. The predictive capacity, efficacy and diagnostic yield of PPCS have been demonstrated to be high with the accepted European protocol suggesting PPCS in athletes should be undertaken every 1–2 years. This includes a questionnaire (to capture demographics including quantifying physical activity and exercise levels, symptoms and family history), a physical examination and a 12-lead electrocardiogram (ECG) [15]. Figure 1 highlights normal athletic criteria on the 12-lead ECG and the abnormal criteria that should trigger follow-up secondary investigations [16]. In this setting, echocardiography is used as a secondary investigation and as such is advocated in the presence of abnormal findings.

It has been suggested that up to 16% of SCD cases are related to structural heart disease with no accompanying electrocardiographic abnormalities [17] and hence some professional sporting organisations also integrate echocardiography as a primary investigation tool and /or a once-only echocardiogram at the athlete’s first assessment (often in adolescence) [18]. The added value of echocardiography at this time point allows the exclusion of congenital, valvular and structural heart disease with evidence to suggest that echocardiography provides additional diagnostic yield in 1.2%—4.5% of adolescent athletes [2, 19]. That aside, based on the challenges of differentiating physiological from pathological adaptation, echocardiography may still provide inconclusive findings and hence further assessment including ESE, 3DE, cardiac magnetic resonance imaging (cMRI) and computed tomography (CT) should be considered and may provide additional value in these challenging cases [20, 21].

**Fig. 2:**
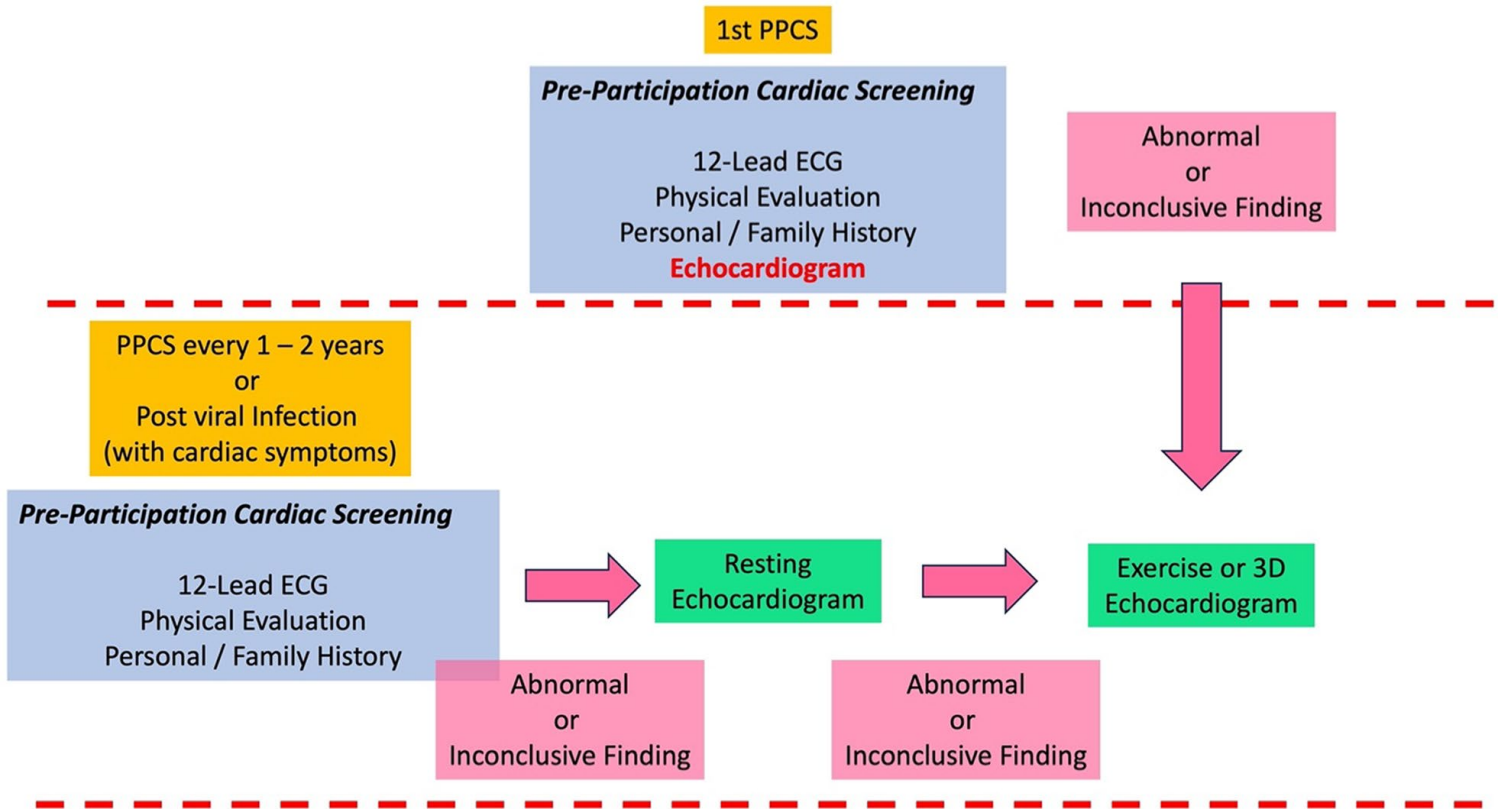
The recommended role of Echocardiography in the Assessment of the Athletes Heart

There are data highlighting the small risk (1–4%) of (peri)myocarditis following viral infections (including COVID-19) in athletic populations [22] with a higher prevalence in symptomatic individuals or those experiencing a debilitating illness [9]. Based on this, the role of echocardiography in the return to play of athletes recovering from viral infection has received appropriate attention [23, 24]. Although the details of the ‘return to play’ training protocols may vary, there is a consensus that an athlete with cardiac symptoms would benefit from an echocardiogram before returning to physical activity [25]. The echocardiogram is aimed at confirming normal ventricular structure and function and excluding any evidence of pericardial or myocardial involvement [25]. Figure 2 highlights the recommended role of echocardiography in the assessment of the athlete’s heart.

### The athletes heart

Our understanding of the athlete’s heart has developed significantly over recent years in tandem with the evolution of echocardiographic techniques and technology [26]. The echocardiographer involved in the assessment of an athlete should understand 1) the physiological adaptation of cardiac structure and function that occurs in response to regular exercise training, 2) the variability depending on the type and volume of training and 3) the impact of the athlete’s ethnicity, sex, age and body size. The following section aims to summarise these key points, and we draw the reader’s attention to the narrative review [26] detailing the multi-factorial aspects associated with physiological cardiac remodelling.

It is well established that exercise training causes adaptation of the heart linked to improvements in cardio-respiratory fitness [27]. Hence an individual athlete’s training volume (defined as training intensity x training duration or Metabolic Equivalent Test (MET hrs/wk = MET × training duration)) should be estimated. An example of a select range of sporting disciplines and their specific METS is highlighted in Table 1. In summary, low- intensity exercise is defined as corresponding to 1.8–2.9 METS, moderate intensity is defined as 3–6 METS and high-intensity exercise as > 6 METS [28].

**Table 1:** Sporting examples demonstrating specific MET values for determining exercise Intensity (adapted from Hermann et al. 2023)

Sporting discipline	Metabolic equivalent (MET)
Soccer	9.5
Running (6 mph)	9.3
Running (7.5 mph)	11.0
Running (8 mph)	12.0
Cycling (Racing > 20 mph)	16.8
Cycling (Leisure < 10 mph)	5.8
Cricket	4.8
Rugby	8.3
Tennis (singles)	8.0
Field Hockey	7.8
Netball	7.0
Boxing	12.3
Golf	4.5
Rowing (competitive)	15.5
Swimming (leisure)	6.0
Swimming (competitive)	10.5
Weight lifting	6.0

The magnitude of adaptation is greatest in those athletes who engage in predominantly mixed (combined dynamic and strength) or endurance exercise rather than pure resistance training [29–31]. Endurance/ dynamic activity can be defined as aerobic isotonic dynamic exercise at incremental workloads of 70–90% of maximum heart rate and includes sporting disciplines such as long- and middle-distance running, swimming or cycling as well as common team sports such as soccer and basketball. Resistance training can be defined as anaerobic dynamic exercise at incremental workloads of > 30% maximal voluntary contraction and includes sporting disciplines such as martial arts, sumo wrestling, shot-put and weightlifting. It is important to note that many sporting disciplines and training regimes involve a combination of resistance and endurance exercise (for example, boxing, rugby, rowing, and American football).

Studies using echocardiography have highlighted the specific type of left ventricular (LV) structural adaptation in athletes and have usually highlighted normal geometry or eccentric hypertrophy i.e. LV cavity enlargement with concomitant increase in LV wall thickness [32]. Left ventricular concentric hypertrophy is rare in all types of training (3–12% and 0–8% in male and female athletes respectively [32, 33]) and although it has been documented more frequently in athletes of black ethnicity and those who are older with a lifelong exposure to exercise [32–34] there is little evidence to support previous theories of it being a primary response to resistance training [27, 35]. Most studies highlight the multifactorial nature of the athlete’s heart with additional factors such as body size [32] also impacting the magnitude of structural adaptation [26]. In view of this, scaling to body size is mandated in athletes. Although the ideal approach is to scale allometrically, there is currently a lack of sufficient population-based exponents and the associated normal athletic ranges to make this approach clinically useful and hence a standard ratiometric / linear scaling to body surface area (BSA) or height is currently recommended.

Structural adaptation of the right ventricle (RV) occurs in athletes of predominantly mixed / endurance exercise training [36, 37] with balanced adaptation of the inflow and outflow [38]. These adaptations occur irrespective of body size [39] and ethnicity [40]. Right ventricular adaptation [40, 41] is also proportional to LV enlargement and hence an objective and subjective assessment of the relative adaptation can provide valuable insight [42]. The atria of the heart are no exception to physiological enlargement with the greatest magnitude of adaptation seen in athletes of high dynamic sports [43], balanced to both right and left atria and across athlete demographics [44, 45]. Although absolute values of all chambers are greater in males, female athletes also demonstrate proportional adaptation highlighting the independent impact of a training stimulus.

The echocardiographic assessment of LV and RV function (systolic and diastolic) is important to aid differentiation from inherited cardiomyopathies and hence a thorough understanding of functional adaptation to exercise training is vital [26]. Generally, systolic functional indices, ejection fraction (EF), global longitudinal strain (GLS), RV free wall strain (RVFWS) RV fractional area change (RVFAC) and tissue Doppler indices are within the published normal ranges for non-athletes [26]. However, it is important to note that 12–17% of predominantly male athletes may have borderline-low or reduced resting values of EF, GLS and RVFAC [20, 46–49]. The mechanism is thought to be related to the physiologically enlarged chambers requiring a lower contractile state to deliver a sufficient stroke volume (SV) at rest alongside a low resting heart rate (HR) [50] secondary to high vagal tone. It is important to note that these physiological functional changes are related to structural adaptation and hence it is less common to observe low resting functional indices from normal-sized chambers such as in resistance trained athletes [27, 51, 52] There are a small proportion of athletes with low resting function that will have a genetic predisposition for dilated cardiomyopathy [47] and hence we encourage echocardiographers to have a low threshold for recommending ESE as an additional assessment in this setting (see Fig. 2).

Left ventricular diastolic function is superior in the athlete, related to high training volumes and high cardio-respiratory fitness [53]. This is achieved through enhanced relaxation, faster reduction in LV pressures and greater untwist which are likely to be more apparent during exercise when increased SV is required. At rest the athlete will typically exhibit normal or supranormal indices of diastolic function with only 1.8% of athletes displaying lower values than what is considered normal for non-athletes [54]. The presence of diastolic dysfunction warrants further investigation. Atrial function is fast becoming an important parameter in the echocardiographic assessment of those non-athletic individuals with LA enlargement and diastolic dysfunction [55]. In view of this, LA and RA strain may have additional diagnostic value as studies have demonstrated that athletic physiological adaptation presents with normal values of reservoir, conduit and booster LA and RA strain irrespective of atrial size [43].

There is evidence to suggest that the morphology of the aortic root in young athletes remains normal [56] but is larger when compared to non-athletes [57]. The greatest dimensions are seen in high-dynamic endurance male athletes [57, 58] but with values above established absolute cut-offs (40 mm in male athletes and 38mm in female athletes) seen in only 0.2% and 0.4% respectively [57]. The diameter of the aortic root is linearly related to the height of the athlete [56] and therefore it is important, as with other cardiac indices, to scale appropriately. It is noteworthy that these values do not apply to master endurance athletes aged 50–70 years old of whom an aortic root diameter exceeds 40mm in 31% males and 8% females [41].

**Fig. 3:**
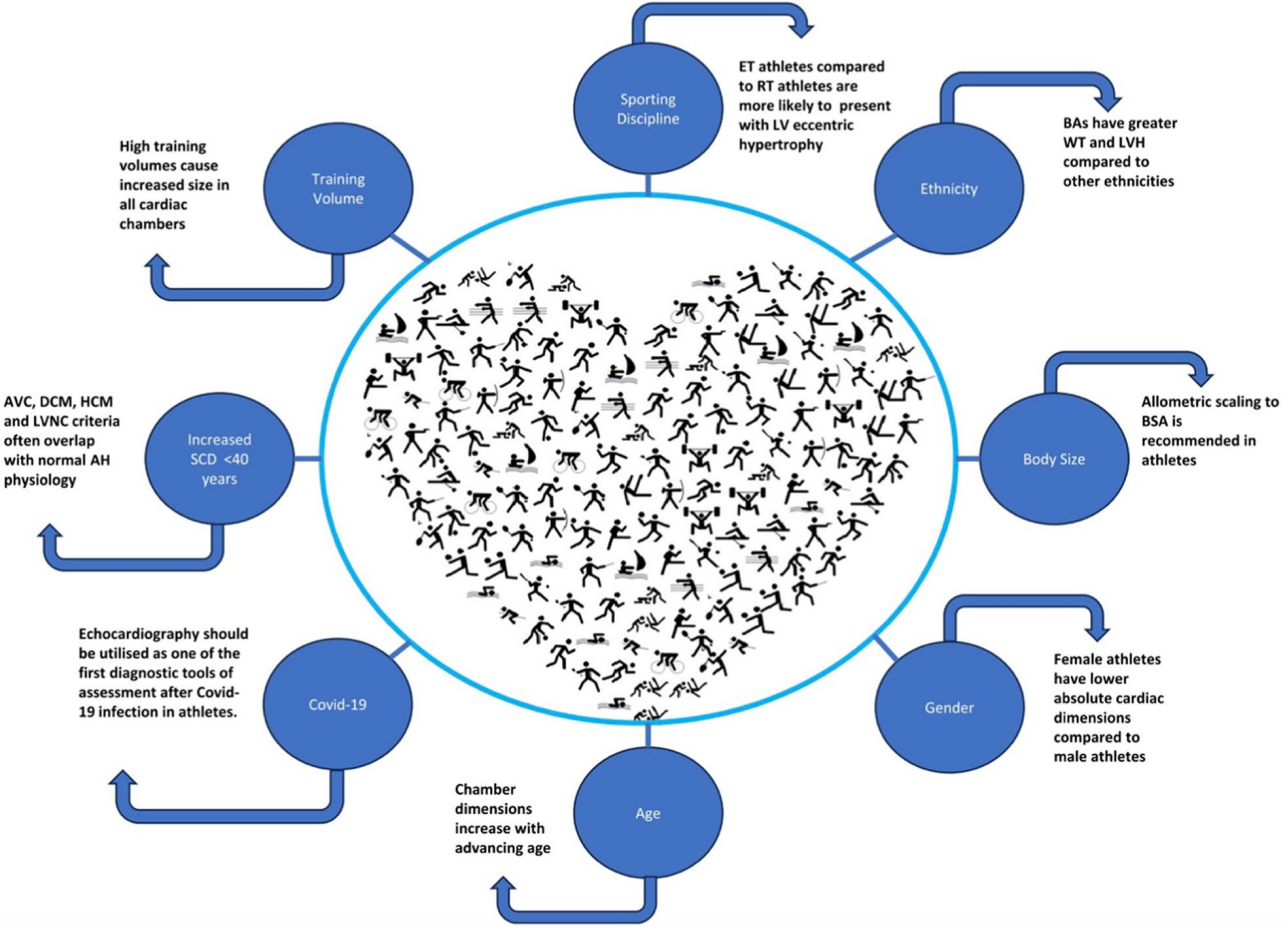
The multi-factorial nature of the athlete’s heart—an overview

Table 2 and Fig. 3 have been adapted from Flanagan et al. 2023 [26] and summarise the multifactorial nature of the athlete’s heart.

**Table 2:** The multi-factorial nature of the athlete heart—points for considerations

Sporting type	Endurance athletes often present with physiological eccentric hypertrophy of the LV; however concentric remodelling in any athlete is rare Endurance athletes typically present normal or decreased systolic function and normal or superior diastolic function compared with the non-athletic population Athletes engaging in endurance disciplines may present with bi-atrial dilatation which is strongly correlated with exercise capacity Endurance and resistance trained athletes tend to present larger aortic root size than the non-athletic population. However, these differences in size are not clinically significant and therefore the presence of a dilated aortic root warrants further investigation
Ethnicity	There is a greater prevalence of LVH in black athletes compared to white, Asian, Arab, Pacific Islander and mixed ethnicity athletes Mixed ethnicity athletes of black lineage have phenotypical similarities to black athletes although less pronounced and have a greater LV wall thickness than white athletes Male black athletes may have a RWT of up to 0.51 or 0.48 in female black athletes, but this finding should be interpreted with caution in the presence of symptoms or a family history of SCD, or an abnormal 12-lead ECG demonstrating, for example, lateral T wave inversion RV structural adaptation is similar across ethnicities Black athletes appear to have larger LA dimensions than white athletes but until this has been reproduced in future studies the existing normal ranges should be applied
Body size	Indexing LV end diastolic diameter and LV mass to fat free mass is most optimal compared to BSA, body mass and height and is important in athletes displaying extreme anthropometry Indexing all chambers to BSA with population-specific allometric *b* exponents, is optimal, however, until these values have been validated across cohorts it is recommended that standard linear scaling to BSA is undertaken in line with BSE guidelines for non-athletes whilst acknowledging the limitations of this approach Indexed aortic root dimensions should be ratiometrically scaled to height. As aortic root dimension values typically fall within established normal limits, indexed aortic reference values may be helpful in the early detection of aortic pathologies in athletes who exceed these limits
Sex	Concentric LVH is extremely rare in female athletes and rare in male athletes Female athletes present with smaller LV, RV and bi-atrial structural dimensions compared to male athletes Males have larger aortic root dimensions compared to females with values of ≥ 40 mm for males and ≥ 38 mm for females being extremely rare. Values exceeding these limits may be indicative of pathology and further assessment would prove beneficial
Age	LV dimensions in adolescent athletes are larger when compared with non-athlete controls. LV cavity enlargement very rarely exceeds 60mm but in cases where it does, whilst also in the presence of an impairment of systolic or diastolic function, a diagnosis of DCM should be considered Adolescent athletes present bi-atrial remodelling compared to non-athlete controls. However bi-atrial function is preserved with LA and RA EF similar between athletes and controls and thus signifies normal physiological remodelling Aortic dilation is rare in adolescent athletes. The aortic diameter cut off values of 40mm for males and 38 mm for females may not be appropriate for the adolescent athlete and therefore scaling to height is warranted

## The standard echocardiographic examination

### Pre-echocardiographic information

Whether as a screening tool or a secondary investigation, echocardiography aims to identify underlying inherited or congenital structural cardiac disease. Therefore, the echocardiographer needs to be knowledgeable about these conditions and proficient in detecting or excluding pathology. Additionally, the echocardiographer must recognise and acknowledge uncertainties that may require further tailored downstream investigations. It is equally important that they fully understand the complexities and nuances associated with the normal athlete’s heart. In all settings, the echocardiogram should be used alongside a 12-lead ECG, a questionnaire (to capture symptoms, family history and training volume), and, in some settings a physical examination [15]. The information obtained will influence the scope and focus of the echocardiographic examination. For example, an athlete aged over 16 years presenting with non-training related 12-lead ECG changes such as T-wave inversion in the anterior leads would warrant a closer assessment of the RV and septal defects. In contrast, T-wave inversion in the lateral leads, regardless of age, would warrant similar scrutiny of the LV apex and broader phenotypic features of an underlying cardiomyopathic substrate. In addition, an understanding of the athlete’s ethnicity, sex, body size, age, training type and volume will provide insight into the expected type and magnitude of athletic adaptation (see Tables 1 and 2 and Fig. 3). The pre-echocardiographic information will contextualise and contribute to the subsequent management of the athlete, and therefore, it is recommended that, wherever possible, this information is readily available before the examination.

The athlete should attend the examination having not trained for a minimum of 3 h prior. There is strong evidence demonstrating acute effects of high intensity and endurance exercise on LV and RV systolic and diastolic function. These effects include a transient reduction in EF, E velocity and GLS with an increase in RV size with reduced RVFAC / RV strain [59–61]. These studies highlight the physiological nature of this phenomenon and generally demonstrate a return to baseline values within 3 h.

It is apparent that the cardiac chambers will adapt differently during a competitive season, with more marked physiological remodelling occurring during those periods of higher training volume [62–65]. As a result, the potential for a broader diagnostic grey area exists for both one-off and serial assessments. Due to the inherent logistics associated with sports practice it is challenging to standardise the timing of a cardiac assessment. Therefore, seasonal-linked variation in cardiac adaptation should be an expected phenomenon and prompt and reinforce the echocardiographer to establish the athlete’s past and current training load and subsequently interpret accordingly.

The current use or past use of performance-enhancing drugs should be noted where the athlete divulges this information or where there is a suspicion. In those athletes who are currently using Androgenic Anabolic Steroids (AAS) it is common to find increased LV mass beyond the values that would be expected for their training volume, type and body size [66]. This often manifests as a balanced increase in cavity size and wall thickness (eccentric hypertrophy) with reduced EF, GLS and left atrial function [67–69]. The previous use of AAS has been shown to have a lasting impact on cardiac size and function albeit at a lower magnitude than those athletes currently using [69].

### The echocardiographic examination

**Fig. 4:**
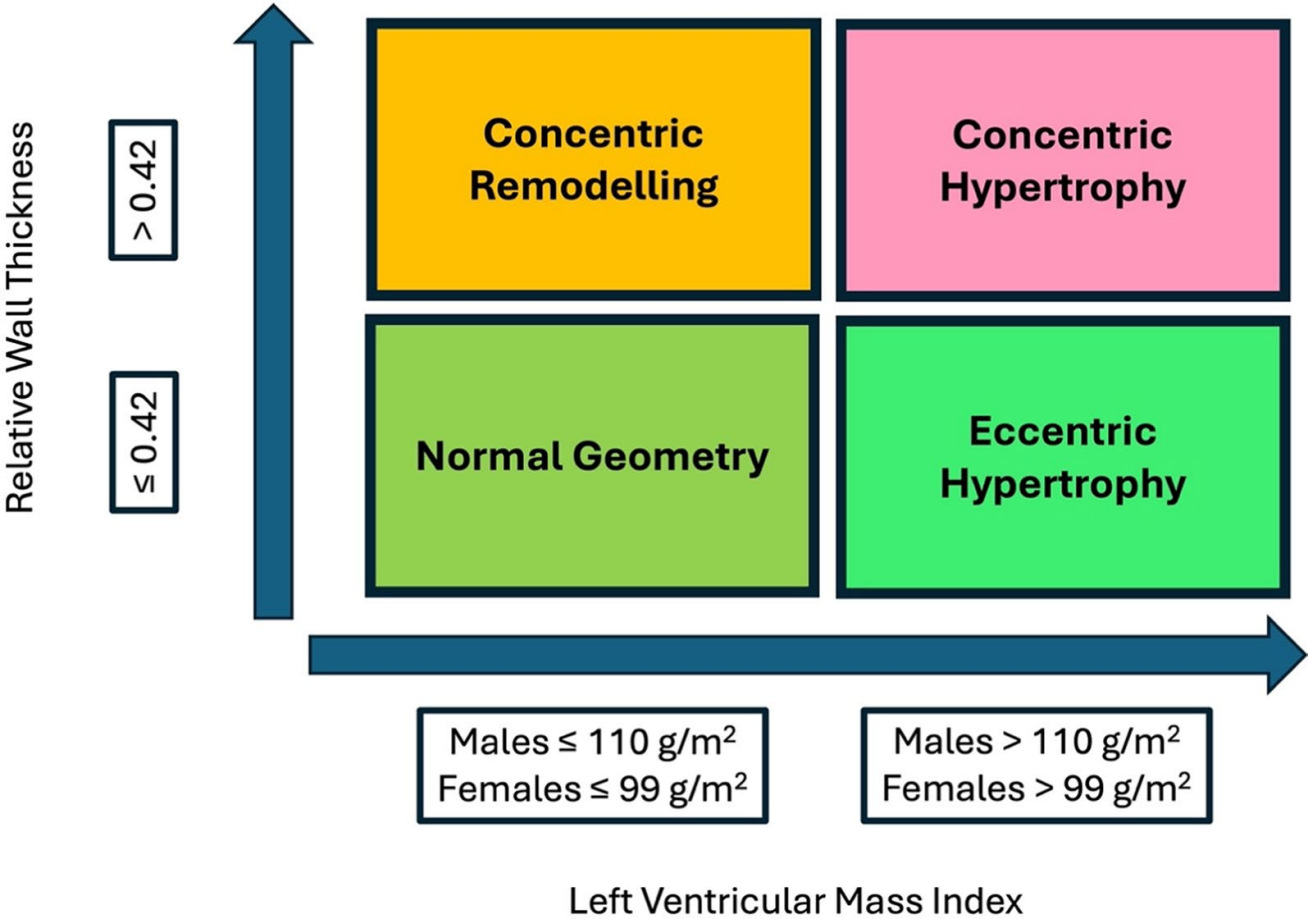
Defining LV Geometry using Standard 2-dimensional Echocardiography

**Fig. 5:**
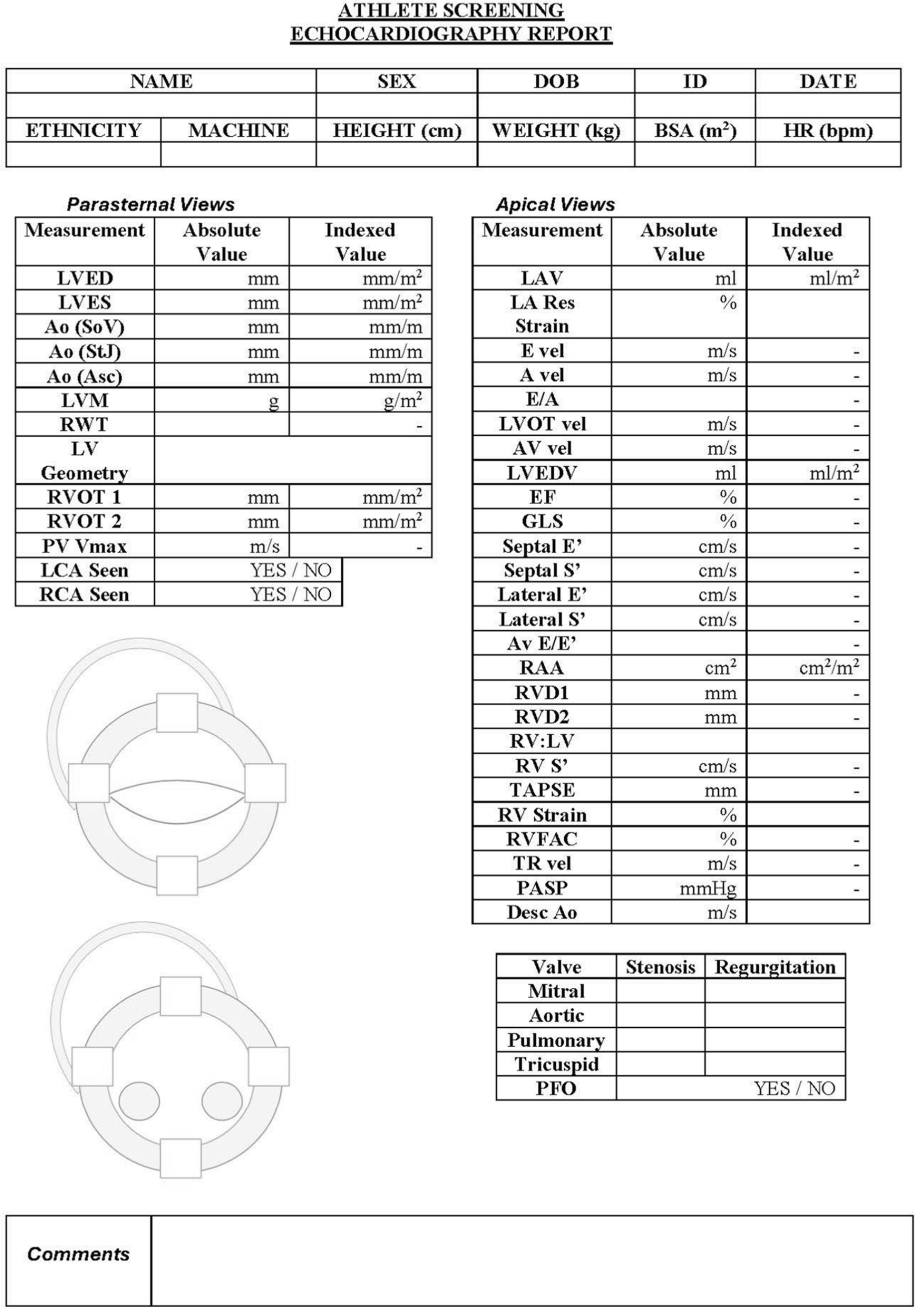
Exemplar Echocardiographic Report

The echocardiogram will be directed by the pre-echocardiographic information but should also fully adhere to the BSE minimum dataset [70]. The BSE minimum dataset is subsequently extended to 1) include additional parasternal short axis (PSAX) images to accurately define LV wall thicknesses from eight myocardial regions (basal and mid anteroseptum, inferoseptum, inferolateral and lateral walls), 2) provide detail of the extent of myocardial trabeculations, 3) demonstrate the origin of both the left and right coronary arteries and 4) include GLS where possible. The inclusion of the maximal wall thicknesses should replace any wall thickness measurements made in a parasternal long-axis (PLAX) orientation and the anteroseptal and inferolateral values should be used to calculate the LV mass and relative wall thickness (RWT). A geometry classification should be offered as per standard reference values for non-athletes [71] (see Fig. 4) and it is important to provide absolute and scaled chamber dimensions, volumes and areas to BSA (aortic dimensions scaled to height). In addition, the apical views (AP4CH, AP3CH and AP2CH) should be optimised to provide technically adequate images for the post-processing assessment of GLS, and all functional indices should be clearly defined including the use of Simpsons biplane or 3DE methods for LV volumes and LV EF. Table 3 highlights the extended protocol with examples of the required images and any technical considerations. Figure 5 provides an exemplar reporting template highlighting the required measurements. During the examination, it may become appropriate to draw on other guidelines including but not limited to the following references [72–77]. The subsequent sections in this guideline will highlight the additional echocardiographic insight required to aid the differentiation between physiological and pathological adaptation.

**Table 3:** Extended athlete’s heart echocardiographic protocol

View and Modality	Explanatory Note	Image
PSAX (Aortic Valve Level)	(1) Identify coronary ostia. The left and right ostia usually originate from their respective aortic sinuses (2) Ensure origin is at sinus level (3) Identify proximal courses and aim to exclude aberrant vessel, especially malignant course between great vessels (aorta and pulmonary artery)	
PSAX (Basal LV Level)	LV wall thicknesses should be measured from the maximum dimension at end diastole from: (1) Anterior septum (2) Inferior septum (3) Posterior/Inferolateral wall (4) Lateral/Anterolateral wall	
PSAX (Mid LV Level)	LV wall thicknesses should be measured from the maximum dimension at end diastole from: (1) Anterior septum (2) Inferior septum (3) Posterior/Inferolateral wall (4) Lateral/Anterolateral wall	
PSAX (Mid to apical LV level)	(1) Excess LV trabeculation is a common finding in athletes (2) LV hypertrabeculation is more prevalent in black athletes (3) Red-flags—thinned compacted layer <5 mm and regional wall motion abnormality in the region of excess trabeculation. Further imaging is advised to exclude LV hypertrabeculation/cardiomyopathy	
AP4CH, AP2CH and AP3CH for GLS	Provide GLS where possible. Bear in mind the technical limitations including image quality, foreshortening and maintaining frame-rates as high as possible up to 90 frames per second

### Image and data interpretation

We have provided a consensus of echocardiographic ‘normal ranges’ for chamber size and function in males and females (white and black athletes where available) derived from key studies and meta-analyses (see Table 4). These should be used as a guide and interpreted within the context of the individual athlete’s training type and volume. For example, a pure resistance-trained athlete of average body size (or one with low training volume defined by their METS x duration [see Table 1]) is unlikely to have chamber sizes close to the upper cut-offs, whilst an endurance athlete with a high training volume is more likely to reach values close to the cut-offs. More so, an athlete with a smaller chamber size or higher heart rate is less likely to have functional parameters close to or below the normal range. The balanced nature of physiological adaptation and our understanding of proportionality should also be considered such that the RV:LV ratio is infrequently greater than 0.9 [37], atrial dilatation often occurs alongside other chamber enlargement [39, 78] and the RVOT1:RVD1 ratio rarely exceeds 1.0 [79]. It is also important to note that body size impacts cardiac size and therefore in the extremes of anthropometry, absolute chamber values may fall outside of the normal range justifying the need to include scaled values [80].

**Table 4:** Consensus of Upper / Lower Range for Chamber Structure and Function in Athletes over 18 years of Age

Parameter	Male	Male BA	Female	Female BA
Left ventricle, left atrium and aortic root				
LVEDd (mm) [81–83]	63	62	56	56
LVEDd index (mm/m^2^) [81]	33	–	34	
IVSd (mm) [81–83]	12	15	11	12
PWd (mm) [81–83]	12	15	11	12
LVM (g) [32, 81–83]	271	442	171	271
LVMi (g/m^2^) [33, 34]	143	153	117	116
RWT [34]	0.44	0.51	0.43	0.48
LVEDVi (ml/m^2^) [81]	103	–	91	–
EF (%) [81]	47	–	47	–
GLS (%) [51, 84, 85]	− 16	–	− 16	–
Septal E’ (cm/s) [54]	7.7	–	7.7	–
Lateral E’ (cm/s) [54]	10.6	–	10.6	–
LAVi (ml/m^2^) [78]	40	–	34	–
Aortic Root (SoV) (mm) [56, 57, 86]	39	–	33	–
Aortic Root index (SoV) (mm/m) [56, 58]	20	–	19	–
Right ventricle and right atrium				
RVD1 (mm) [36, 37, 40]	54	55	50	46
RVD1 index (mm/m^2^) [36, 37, 40]	28	27	28	27
RVD2 (mm) [36, 37, 40]	46	48	43	42
RVD2 index (mm/m^2^) [36, 37, 40]	24	25	25	25
RVD area (mm) [36, 37, 40]	40	38	33	29
RVD area index (cm^2^/m^2^) [36, 37, 40]	20	19	18	17
RA area (cm^2^) [36, 37, 40]	29	27	25	21
RVOTplax (mm) [40]	40	39	38	34
RVOTplax index (mm/m^2^) [40]	20	19	22	20
RVOT1 (mm) [40]	43	43	41	37
RVOT1 index (mm/m^2^) [40]	23	21	23	22
RVOT2 (mm) [40]	34	31	29	28
RVOT2 (mm/m^2^) [40]	18	15	16	16
RVOT1/RVD1 [79]	1	–	1	–
RV:LV [79]	1	–	1	–
TAPSE (mm) [37]	17	–	17	–
RVFAC (%) [36, 37]	32	–	32	–
RV S’ (cm/s) [37]	10	–	10	–

**Fig. 6:**
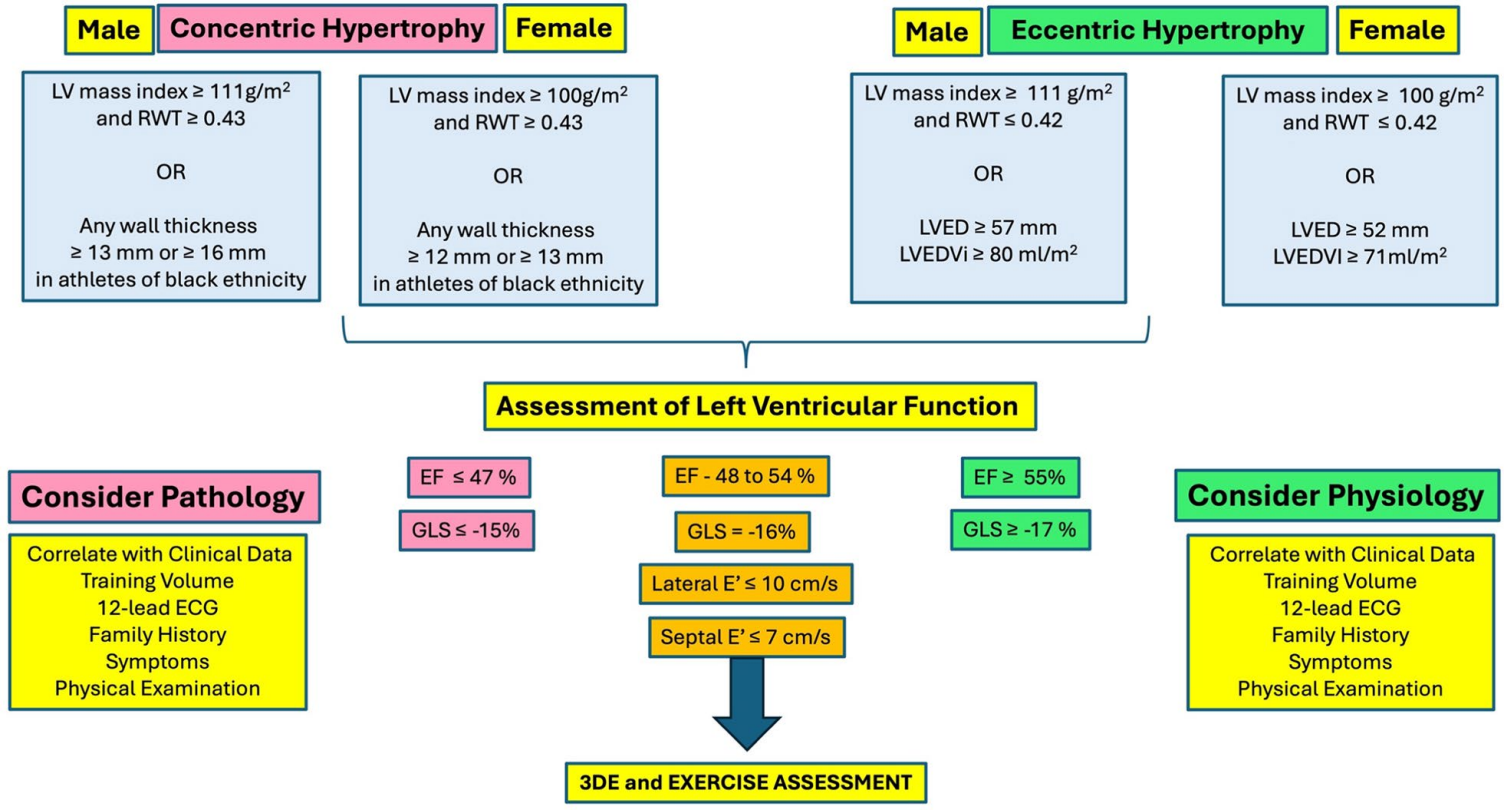
Left Ventricular Interpretation Algorithm

**Fig. 7:**
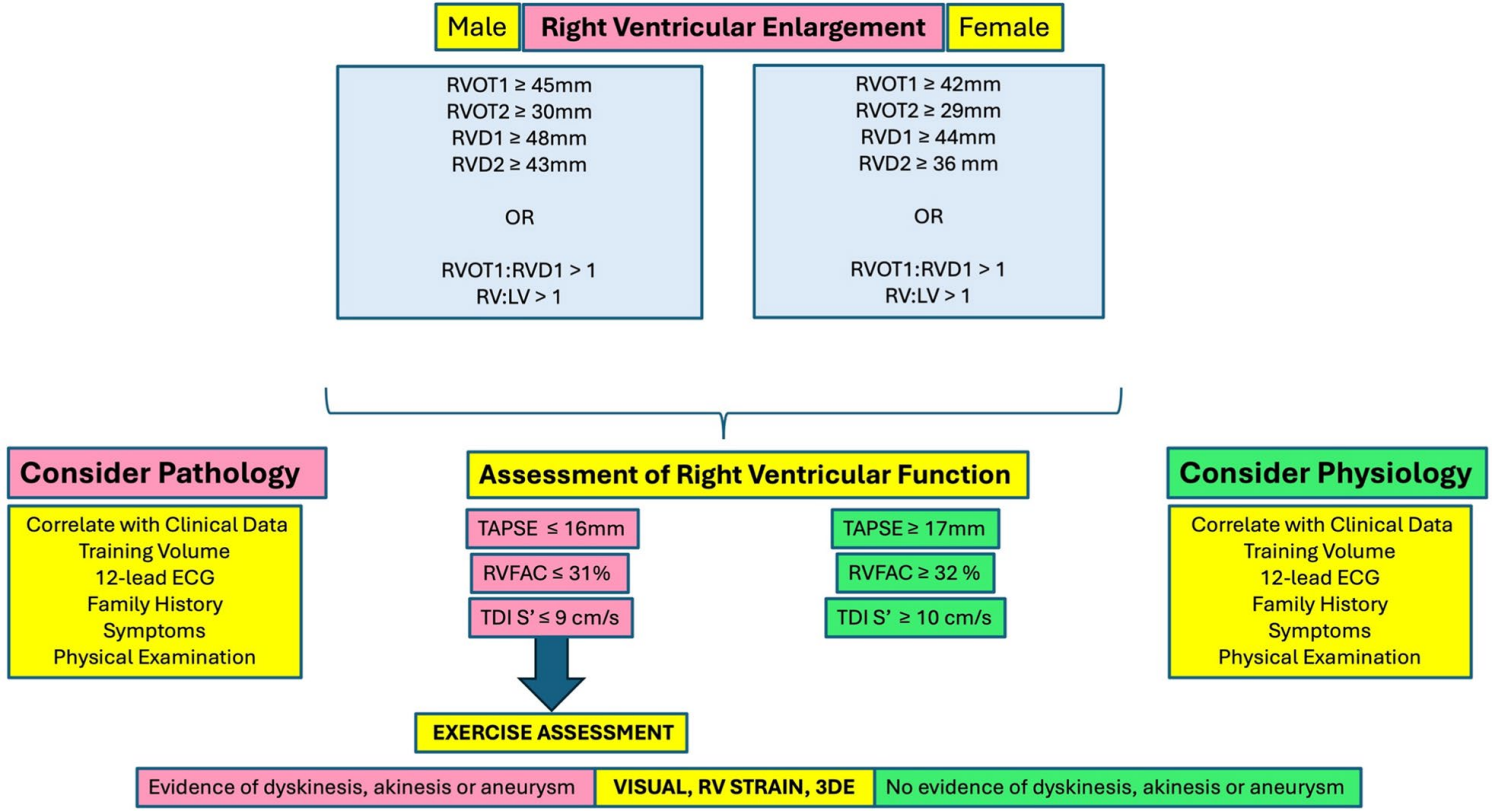
Right Ventricular Interpretation Algorithm

The following two algorithms have been proposed to support the interpretation of the echocardiographic LV and RV data and provide recommendations for the onward management of the athlete (see Figs. 6 and 7). Note that entry level into the LV algorithm draws upon the BSE non-athlete cut-offs whilst entry into the RV algorithm is based on a combination of both BSE non-athlete cut-offs and our athlete consensus data reported in these guidelines and is based on absolute values only. This conservative approach is aimed at maximising the sensitivity of the algorithms whilst maintaining specificity (due to the heterogeneous presentation of the athlete’s heart) and hence reducing the risk of false negative findings. The algorithms do not include the use of RV or LA strain due to the limited athlete data available however their use is not discouraged and in fact, may provide added value to conventional parameters when considering referral for additional assessments/ investigations.

### The left ventricle

The LV algorithm starts with the definition of hypertrophy as per standard geometry classifications or the presence of an increased wall thickness/ cavity size [71]. Based on the relatively infrequent presentation of concentric hypertrophy in athletes [32], if encountered, the echocardiographer should already be considering pathology, whereas if the athlete presents with eccentric hypertrophy the echocardiographer should primarily consider physiology. In both instances, it is important to place the findings in context by considering training type and volume, body size and any clinical correlates. Irrespective, a full functional assessment is important to further refine the likelihood of pathological or physiological adaptation and is aimed at providing valuable information into the grey area of cardiac adaptation. The presence of an LV EF ≤ 47% or GLS ≤ − 15% should raise a red flag for potential pathology whereas an LV EF ≥ 55% or GLS ≥ − 17% is more likely to represent physiology. The intermediate range including reduced diastolic function and/or LV EF between 48 and 54% and GLS of −16% may warrant additional assessment with ESE [20].

### The right ventricle

The RV algorithm starts with the criteria for RV enlargement at either outflow or inflow or the presence of disproportionate RV inflow to outflow or RV to LV ratios. Where enlargement exists, it is essential to further put this into the context of the athletes training type and volume and body size and to utilise an assessment of RV function. In the presence of reduced longitudinal function (S’ ≤ 9 cm/s and/or ≤ TAPSE 16 mm) or reduced RVFAC (≤ 31%) then additional assessment with ESE is indicated [87]. A comprehensive subjective assessment should be applied to determine the presence or absence of regional wall motion abnormalities or aneurysms. This may be supported using RV strain or 3DE; however, our limited understanding of normal athlete regional values suggests that it should not be relied upon in isolation. If wall motion abnormalities or aneurysms are identified on examination, then it is extremely important to initiate the cascade for further assessment.

### The atria and aortic root

Dilatation of both atria is frequently observed in athletes across a range of demographics with the magnitude being proportional to training volume and cardiorespiratory fitness [88, 89]. The dilatation is unlikely to be isolated (albeit it can be) and tends to co-exist with ventricular enlargement [90]. Atrial enlargement, defined by an indexed volume, should be interpreted as ‘normal physiological enlargement’ when there is no evidence of significant valve disease or ventricular diastolic dysfunction.

The aortic root should be measured at the sinus of Valsalva, sinotubular junction and proximal ascending aorta and should be presented as an absolute value as well as scaled to height [71]. The value should fall within the normal range for non-athletes, and therefore any deviation from these should be interpreted as abnormal [57, 91]. It is important, to note effacement or relative dilatation, particularly in the presence of a bicuspid aortic valve.

### The report

The echocardiographic report should follow local guidelines but with the interpretation relative to the individual athlete. Left and right ventricular geometry, size and function should be described and interpreted in the context of the athlete’s demographics. The presence or absence of congenital and valve disease should be highlighted, the size and morphology of the aorta should be described and the identification (or failure to identify) of the coronary ostia should be stated. A conclusion should be offered stating whether the findings are consistent with physiological adaptation or whether further investigations are recommended.

## Differentiation from disease

The following section highlights the nature of the cardiac phenotype specific to the athlete in various cardiac conditions whilst highlighting the challenging grey area of diagnosis. Please also refer to the relevant BSE disease-specific guidelines.

### Hypertrophic cardiomyopathy

Hypertrophic cardiomyopathy is an autosomal dominant cardiac myocyte disease characterised by LVH, diastolic dysfunction, dynamic LV outflow obstruction and myocardial ischaemia. Between 30 and 60% of patients have been found to have a pathogenic variant in genes encoding sarcomeric contractile proteins [92]. Left ventricular hypertrophy is required for a clinical diagnosis: ≥ 15 mm in at least one LV myocardial segment in the absence of abnormal loading conditions, or ≥ 13 mm in the context of a pathogenic variant or family history [93].

The prevalence of clinical HCM in adult populations has been reported to be 1 in 500, with the UK biobank population suggesting a general population prevalence of LVH ≥ 15 mm in 0.11% [94]. The incidence of SCD across a large series of reported groups was 0.43% per annum (95% CI 0.37–0.50%), but more pertinently this was reported as 1.09% (95% CI 0.69–1.73%) per annum in young adults and children [95]. In young athletes, HCM is recognised as a common cause of SCD [4, 5, 7].

Echocardiography is typically the first line imaging tool to diagnose and assess severity of HCM. Left ventricular EF is typically normal/supranormal in HCM, until “end stage” disease when there is a deterioration but reduced GLS can be seen in HCM with normal LVEF [96]. In athletes, GLS ≤ 15% is suggestive of pathological LVH [51, 85, 97].

Additional anatomical/ macroscopic features suggestive of a HCM phenotype may include abnormal mitral valve (MV) and sub-valvular apparatus [98, 99] which can contribute to systolic anterior motion of the MV and outflow tract obstruction in 30–50% of affected individuals, a feature not seen in athletic adaptation. Individuals with HCM can demonstrate abnormal elongation of the MV leaflets (anterior MV leaflet > 30 mm, posterior MV leaflet height > 15 mm), abnormal papillary muscles (hypertrophy and apical displacement), and abnormalities of the chordae including direct insertion onto the anterior MV leaflet and elongation that may result in prolapse. The additional presence of LV crypts also warrants further investigation.

A minority of athletes exhibit physiological LVH that falls within the so called ‘grey zone’ (i.e. 13–15 mm). Studies have attempted to differentiate physiological LVH from mild HCM [100, 101] and identified the main discriminators suggestive of HCM in an athlete as LVED ≤ 53 mm, LA ≤ 39 mm, abnormal diastolic function, concentric LV geometry and asymmetrical LVH (IVS:PW ratio ≤ 1.3). It is important to acknowledge that these discriminators have been based on comparisons between healthy athletes versus sedentary HCM patients. In comparison with sedentary HCM patients, athletes with HCM demonstrate a lesser magnitude of LVH (often 15-16mm), larger LV cavity dimensions and higher diastolic indices. There is also a lower prevalence of outflow tract obstruction and valve disease.

Athletes with HCM can demonstrate co-existent physiological adaptation. In a comparison between athletes with HCM and healthy athletes with ‘grey zone’ LVH, LA diameter was a poor discriminator, with 87% of athletes with HCM demonstrating normal LA dimensions. Additionally, 14% of athletes with HCM demonstrated LVEDD > 54 mm [54, 102]. ROC analysis identified LVED < 51 mm and septal E’ ≤ 11 cm/s as the only positive predictors for HCM. It is important to note that based on the heterogeneous nature of both the athlete’s heart and HCM phenotypes, the external validity of these findings is limited.

### Dilated cardiomyopathy

Four chamber dilation is the hallmark of endurance athletes but is also noted in athletes participating in sports with a high dynamic exercise component, such as soccer and rugby [103]. High cardiac output, often sustained for hours during training and competition, can lead to marked balanced dilation of both atria and ventricles [104]. The athlete with physiologic dilation of the LV should demonstrate normal or supra-normal indices of diastolic function. Normal trans-mitral pulsed wave and LV TDI in an athlete with LV dilation are consistent with physiologic remodelling [53].

Dilation of the LV is also commonly encountered in patients with DCM. The term DCM refers to a group of aetiologically heterogeneous myocardial disorders defined by LV or biventricular dilation in conjunction with global or regional systolic dysfunction not otherwise explained by abnormal loading conditions such as hypertension, valvular pathology, or coronary artery disease [105]. Common aetiologies of DCM include genetic alterations (i.e. Titin, Lamin A/C mutations), post-infectious sequalae, alcohol toxicity, underlying systemic immune disorders, and tachycardia-induced myopathy. There are a subset of patients with DCM that will present without systolic dysfunction and hence complicating the diagnostic challenge [106].

The differentiation of DCM from benign exercise-induced ventricular dilation requires the integration of exercise training/competition history, clinical presentation, and echocardiographic imaging data. Benign physiologic ventricular dilation often occurs among asymptomatic, high functioning endurance athletes and team athletes who participate in sports with a high endurance component. Dilation of the LV (or RV) in athletes participating in sporting disciplines with a low endurance or low dynamic exercise component should trigger concern for DCM. Similarly, all athletes presenting with impaired exercise capacity, a family history of cardiomyopathy, or malignant ventricular arrhythmias, alongside LV (and/or RV) dilation should be considered at high risk for DCM. Ventricular dimensions are neither sensitive nor specific for DCM [107, 108]. Although formal criteria for DCM include specific cut-points for LV size, there is considerable overlap between ventricular sizes in patients with DCM and highly trained endurance athletes. Ventricular dilation of a single chamber in isolation should never be considered physiologic and should prompt concerns for DCM.

Inherent in the definition of DCM is reduced systolic and diastolic function. The finding of an LV EF less than 40% and/or impaired indices of diastolic function, should raise concern for DCM and prompt a comprehensive diagnostic evaluation. In contrast, a mildly reduced LV EF (45–50%) may be identified among highly trained endurance athletes [109]. As such, the diagnosis of DCM in an otherwise healthy endurance athlete with LV dilation should not be based solely on a mildly reduced LV EF in isolation. Additional features suggesting disease, include isolated enlargement of the LV, myocardial thinning and regional wall motion abnormalities. The presence of impaired GLS < − 17% should also raise the suspicion of DCM albeit the overlap with reduced GLS in athletes further complicates the differentiation. Among endurance athletes with mildly reduced LV EF and/or GLS, assessment of LV systolic function during exercise to confirm normal augmentation is warranted [110] (see ESE section).

### Left ventricular hypertrabeculation

Left ventricular hypertrabeculation in adults is characterised by prominent LV trabeculae, deep intertrabecular recesses and a thin compacted epicardial layer. In some individuals, these changes are associated with progressive LV systolic dysfunction, ventricular arrhythmias and thromboembolic complications [111]. The precise aetiology is unclear. Retarded intrauterine morphogenesis has been postulated, however several mutations in genes implicated in familial cardiomyopathy have also been reported (e.g. in the sarcomeric protein, Z-disc, cytoskeleton and nuclear envelope) [112–116]. The structural phenotype may be stimulated by acquired conditions associated with a high cardiac preload such as severe valvular regurgitation, anaemia, pregnancy or following intensive physical exercise [117–119].

Left ventricular hypertrabeculation is observed in up to 8% of athletes and is generally of no concern [117, 119–122]. Athletes with LV hypertrabeculation might be suspected of having cardiomyopathy if they exhibit cardiac symptoms, report a family history of the disease, show abnormal repolarisation changes on the ECG (e.g. T-wave inversion in the inferolateral leads), demonstrate LV dysfunction with an EF below 45%, possess a compacted myocardial layer less than 5mm at end-diastole, or exhibit diastolic dysfunction [118]. It is typically advised to conduct cMRI to assess for LV fibrosis or thrombi, and to perform ESE to evaluate for contractile reserve and rule out exercise-induced complex arrhythmias in these athletes.

### Arrhythmogenic cardiomyopathy

Arrhythmogenic cardiomyopathy is predominantly caused by pathogenic variants in genes encoding cardiac desmosomal proteins. The population prevalence is estimated at 1:2000–1:500. The condition is characterised by myocardial fibrofatty infiltration, predisposing to ventricular tachyarrhythmias and is a major cause of exercise related SCD in young athletes [7].

The 2010 Modified Task Force Criteria focused exclusively on RV parameters for diagnosis [123], however, concomitant LV involvement is now recognised in most cases. This has led to a change in terminology such that ACM with predominant RV, LV, or biventricular involvement may be preferable to previous nomenclature (‘arrhythmogenic right ventricular cardiomyopathy’ or ‘ARVC’).

The diagnosis of ACM relies primarily on data from resting and ambulatory ECG, imaging investigations, and genetic / familial evaluation, with ‘major’ and ‘minor’ thresholds within each category. Typical electrocardiographic findings include T-wave inversion in the limb or precordial leads, frequent ventricular extrasystoles or complex ventricular arrhythmias. The 2010 criteria identified echocardiographic dilatation of the RVOT (proximal RVOT ≥ 36 mm or ≥ 21 mm/m^2^), or reduction in RVFAC (≤ 33%), *in combination with* RV regional wall motion abnormalities as imaging features of ARVC. Recently proposed diagnostic criteria (the ‘Padua criteria’) acknowledge the biventricular nature of this disease [124]. In addition to stipulating the presence of RV regional wall motion abnormalities, the Padua criteria are extended to include assessment of LV function (using EF or GLS), and inspection for LV wall motion abnormalities also incorporating the detection of myocardial fibrosis using cMRI. There is also evidence of disproportionate RA enlargement in athletes with AVC. Highlighting the added value of RA volume index (RAVi) as a ratio to LAVi (RAVi:LAVi) [125].

Healthy athletes frequently exhibit ventricular dilatation, including that of the RV, and borderline-low/ mildly reduced resting systolic function. Indeed, around half of elite athletes demonstrate RVOT enlargement numerically exceeding major diagnostic thresholds for ACM [40]. Anterior T-wave inversion is also observed in 4% of athletes, resulting in a diagnostic grey area.

Differentiation between ACM and athletic remodelling requires a multifaceted approach [126]. Sinister cardiac symptoms such as syncope, family history of cardiomyopathy or premature sudden death, complex ventricular arrhythmias on ambulatory monitoring or stress testing, myocardial fibrosis on cMRI, or electrocardiographic patterns not considered typical in athletes [16].

The magnitude of structural remodelling should be proportionate to the athlete’s age, gender, size, and sporting discipline (see Fig. 3). Profound remodelling exceeding thresholds in Figs. 6 and 7 should prompt further investigation. Echocardiography should demonstrate balanced, symmetrical cardiac remodelling (RV/LV ratio ≤ 0.9), whilst disproportionate RV enlargement may suggest pathology [126]. Regional wall motion abnormalities should not be present in healthy athletes. This can be assessed visually, although strain assessment (reduced global or segmental strain, or abnormal patterns such as systolic stretch or mechanical dyssynchrony) permits a more objective evaluation [127]. Profoundly reduced RV function (RVFAC ≤ 30%) [126] and impaired RV long axis function (RV S’ < 10 cm/s, or RV free wall strain < 20%) should be considered abnormal [127]. Borderline-low functional indices in either ventricle should augment rapidly during ESE, which also permits assessment for exertional arrhythmias or symptoms.

### Coronary artery anomalies

Coronary artery anomalies encompass a wide range of congenital variations in the origin of a coronary artery from the ascending aorta and/or its subsequent course. Outcomes are highly variable and include benign and incidental findings, to more malignant CAAs associated with exercise related SCD [4, 7, 17]. CAAs have been reported to account for 9% of deaths in athletic adolescent individuals aged between 10 and 19 years, compared to 1% of non-athletic individuals [128].

Anomalies where the coronary artery course runs posterior to the aorta (retroaortic) or anterior to the pulmonary artery (pre-pulmonic) are generally not prone to compression during exercise and thus may be considered benign after comprehensive evaluation. In contrast, anomalies associated with interarterial or intramural course are typically associated with coronary obstruction, malignant ventricular arrhythmia, or myocardial ischaemia. Additionally, a slit-like orifice or acute angle take-off of the coronary artery ostia and proximal course are additional high risk factors [129]. Notably, an anomalous left coronary artery arising from the right sinus of Valsalva (ALCA) and from the pulmonary artery (ALCAPA) have been frequently observed in autopsy studies of SCD victims, suggesting a higher risk of exercise-related sudden death [129–131]. It is noteworthy that an anomalous CA originating from the pulmonary artery (PA) (ALCAPA or ARCAPA) is rare (0.01%), [132] and 85% of cases present in early childhood, whereas, an anomalous aortic origin of a coronary artery (AAOCA) is more likely to be identified in adolescents and adulthood.

Given the association of CAAs with SCD in athletes, it is essential to thoroughly assess the origins of the coronary arteries when evaluating young athletes [75, 133]. However, this is a complex process that demands a systematic approach, adequate time, and significant expertise [75].

For the purposes of optimising image acquisition and diagnostic yield, athlete position and comfort are crucial. Utilise the coronary pre-set if available and ensure the transducer frequency is set for the highest harmonic frequency. From the PLAX, the origin of the right coronary artery (RCA) from the right sinus of Valsalva may be appreciated. This is particularly helpful in identifying high take off RCAs, which arise above the sinotubular junction (STJ). Additionally, a discrete circle in the vicinity of the ascending aorta in the PLAX view, should raise suspicion of an interarterial or intramural course between the great vessels. The optimal view for assessing the ostial of the coronary arteries is the PSAX view. Athletes may need to be rotated further on to their left and the transducer may need to be angled gradually superiorly with gradual modified sweeping motions of the probe. Optimise the image by adjusting the zoom, sector width, and gain. Utilise colour flow mapping, reducing the Nyquist limit to 30 cm/s, which assists in excluding pericardial folds or venous coronary structures that may cause diagnostic dilemmas. Trace the coronary arteries to their origins using the cine/freeze function, focusing on one artery at a time rather than attempting to demonstrate multiple arteries in one image. As a frame of reference, the RCA typically arises at a 11–12 o’ clock position and the left coronary artery (LCA) typically arises at a 3–4 o’clock position. Clockwise probe rotation may visualise the bifurcation of the left coronary to demonstrate the left anterior descending (LAD) and left circumflex (LCx) arteries; and counterclockwise rotation for the RCA. Both standardised and non-standardised views are necessary, and any uncertainty should be acknowledged in the final report (see Table 5). For a comprehensive overview of this topic, we encourage the reader to a recently published practical guide [75].

**Table 5:** Differentiation from disease—key points

Condition	Key Points	Images
Hypertrophic Cardiomyopathy	Echo parameters to suggest pathological LVH in athletes: - LVH > 15mm - Asymmetrical LVH - Concentric LVH geometry - LVEDD < 54mm - GLS < 16% - Abnormal MV and subvalvular apparatus - LVOTO/SAM - LV crypts	
Dilated Cardiomyopathy	The athlete with physiologic dilation of the LV should demonstrate normal or supra-normal indices of diastolic function. An LV EF less than 40% and/or impaired indices of diastolic function, should raise concern for DCM. Among endurance athletes with mildly reduced LV EF, assessment of LV systolic function during exercise to confirm normal augmentation is warranted.	
Left Ventricular Hypertrabeculation	Left ventricular non-compaction is now no longer considered to be a cardiomyopathy and is identified as an isolated phenotypic trait or occurring in combination with systolic dysfunction and ventricular arrhythmias. Athletes with LV hypertrabeculation in the absence of symptoms, family history of cardiomyopathy, abnormal ECG, impaired LV systolic function or complex ventricular arrhythmias can participate in sports of any intensity. Athletes with LV hypertrabeculation and impaired LV systolic function that is consistent with exercise induced cardiac remodelling should follow the recommendations for athletes with dilated cardiomyopathy	
Arrhythmogenic Cardiomyopathy	Most commonly RV enlargement and dysfunction, with regional wall motion abnormalities. LV or biventricular involvement is increasingly recognised. Diagnosis of ACM is complex and requires integration of data from the ECG, ambulatory monitoring, imaging investigations, and genetics. Newer proposed diagnostic criteria have acknowledged LV involvement, and the utility of echocardiographic strain assessment. Pathology should be suspected when there is profound remodelling exceeding thresholds in Figs. 6 and 7, remodelling which is disproportionate to the athlete’s demographic profile (Fig. 3), unbalanced remodelling, impaired long axis function (TDI or strain), or regional wall motional abnormalities in either ventricle. Vigorous augmentation of cardiac function (including the RV) should be seen during exercise echocardiography in healthy athletes. A period of detraining (at least 6 weeks) should result in partial or complete resolution of training-induced structural and function adaptations in the RV and/or LV.	
Coronary Anomalies	Optimise athlete position to acquire standardised and non-standarised views. PLAX view may give clues to low/high take off coronary arteries, intramural course. Emphasis on modified SAX view at AV level; with angulation of probe above the AV and modified sweeping movements with gentle clockwise and anticlockwise rotation. Focus on optimising the image (zoom, sector width, gain, cine/freeze function) to adequately comment on the (a) individual ostia of the coronary arteries from the ascending aorta; and (b) any potential abnormal course of the ostia or proximal segments of the artery.	
Myocarditis (including COVID)	Myocarditis is an important cause of cardiac damage in athletes TDI and Strain play an important role in the identification of regional abnormalities. Athletes diagnosed with myocarditis required evaluation in expert sports cardiology centres. Return to play protocols should be followed to avoid cardiac complications.	
Aortic Root Disease and Bicuspid Aortic Valve	Increased intensity of sport correlates with larger aortic dimensions, but they rarely exceed normal limits even in endurance athletes. Bicuspid aortic valve is the commonest congenital heart defect, present in 1-2% of the population, with one-third developing aortic stenosis, regurgitation, or aortic root dilation. Risk of SCD is related to sudden aortic dissection, accounting for 0.8% of SCD in athletes	
Mitral Valve Prolapse	Arrhythmic MVP is typically associated with myxomatous degeneration and prolapse of both leaflets with a degree of mitral annular disjunction (MAD). An increase in LV end-diastolic or end-systolic dimensions/volumes or reduction in LVEF within a short time-frame and without a change in training type or volume are more the adverse remodelling effects of chronic significant MR in athletes with MVP. Disproportionate dilatation of the LA is more likely related to the degree of MR in athletes with MVP. ateral S’ that peaks in late systole (‘Pickelhaube sign’) and at a velocity > 16 cm/s has been proposed as a marker of greater arrhythmic risk in MVP and MAD.	

Despite these considerations, limitations and uncertainties may exist in assessing higher or lower take-off coronary arteries, mid to distal vessel course/ termination and intracoronary pathology. In such cases, depending on the clinical scenario, other non-invasive imaging modalities like computed tomography coronary angiography (CTCA), cMRI and functional assessments to assess for CV symptoms, myocardial ischaemia and ventricular arrhythmia may be required. Following identification or suspicion of a CAA, management is primarily based on expert-consensus recommendations and a shared decision-making approach [133–135].

### Myocarditis (including COVID-19)

Myocarditis is a non-ischaemic inflammatory heart muscle disorder and may be complicated by cardiac dysfunction and potentially fatal arrhythmias [11]. It is reported to cause 2–7% of all SCDs in young athletes [7]. Viral infection is the most common aetiology, however other recognised causes including illicit drugs such as cocaine [136] or vaccines such as influenza [137] should be considered.

The spectrum of clinical presentation of myocarditis in athletes is variable and ranges from asymptomatic status, self-resolving symptoms such as mild chest discomfort, dyspnoea or fatigue, to more serious clinical sequelae including decompensated heart failure or cardiogenic shock [138]. Cardiac enzyme markers may be elevated. In addition, the ECG may demonstrate variable findings such as ST segment or T waves changes, low QRS voltages, conduction abnormalities, frequent ectopic beats and sustained arrhythmias.

The gold standard method to establish a diagnosis of myocarditis is with endomyocardial biopsy; however, this is an invasive procedure which has low sensitivity as the pattern of inflammation may be patchy and susceptible to sampling of non-inflamed tissue. Cardiac MRI has evolved as a useful and reliable investigation to establish a prompt diagnosis by detection of ventricular dysfunction, myocardial inflammation or scar [139].

Two-dimensional echocardiography is the first line imaging modality. Diagnostic features of acute myocarditis include regional wall motion abnormalities, LV/RV systolic or diastolic dysfunction, ventricular dilatation, pericardial effusion and an increase in LV mass or ventricular wall thickening [140]. While 2D echocardiography may not detect wall motion abnormalities, additional techniques, such as TDI, strain imaging, contrast echocardiography, or 3DE may unmask subtle cardiac function abnormalities.

Tissue Doppler parameters may be abnormal in both athletes and non-athletes with myocarditis [141–143]. Strain imaging may detect early ventricular dysfunction in individuals with acute myocarditis. In a study of non-athletes with acute myocarditis and preserved LVEF, a significant reduction in GLS was demonstrated when compared to healthy controls [144]. Other studies have identified that strain measurements are more sensitive than 2D echocardiography in identifying subtle regional wall motion abnormalities and diagnosing acute myocarditis [144, 145]. Strain analysis can also be useful to assess improvements in ventricular function over time as the inflammatory process resolves. The assessment of strain should therefore be routinely used in patients with suspected or recovering myocarditis.

When 2D images are suboptimal, contrast echocardiography or 3DE can assist in the diagnosis of myocarditis by identifying subtle regional wall motion abnormalities detecting any left ventricular thrombus. In addition, 3D strain is emerging as a promising tool in the diagnosis of patients with acute myocarditis [146].

Undertaking exercise during the active phase of myocarditis may exacerbate the inflammatory response and induce malignant arrhythmias. Any athlete diagnosed with myocarditis, should be advised to refrain from structured exercise in the acute phase of the illness. When advising on the duration of abstinence from high-intensity exercise or assessing fitness for return to competitive sport, affected individuals should be evaluated and closely supervised by physicians with expertise in sports cardiology in conjunction with consensus guidelines [133].

### Aortic root disease and bicuspid aortic valve

Aortic root disease generally relates to pathologic dilation of the aorta which needs to be differentiated from physiological enlargement secondary to exercise. Increased intensity of sport correlates with larger aortic dimensions, however even in endurance athletes who have the largest diameters, very rarely does this enlargement fall outside of normal limits [147, 148]. A meta-analysis showed the aortic root (SoV) was only 3.2 mm larger in athletes compared to controls, but still within normal limits [148].

Bicuspid aortic valve (BAV) is the most common congenital heart defect, present in 1–2% of the population with 30–50% of individuals developing aortic stenosis, regurgitation, or aortic root dilation [149, 150].

Although it is often straight forward to distinguish a bileaflet from trileaflet aortic valve on echocardiography, the true challenge lies in distinguishing physiological aortic dilation from an underlying aortopathy. The risk of SCD in individuals with aortopathy is related to sudden aortic dissection, accounting for 0.8% of SCD in athletes [7]. Aortic dissection becomes increasingly likely with a dilated aorta due to increased shear wall stress on a thinned enlarged aorta, and an underlying wall abnormality in the aorta. This risk of SCD will, therefore, be magnified for those doing high intensity or highly isometric sports with dissection due to increased blood pressure and subsequent wall stress during exercise. In addition, a dilated aortic root can lead to progressive aortic regurgitation due to failure of coaptation of the aortic valve leaflets.

Although there may be subtle clinical signs of BAV at auscultation, this is unreliable and clinical examination rarely forms part of many pre-participation screening protocols. Moreover, aortic root dilation in an athlete is impossible to detect clinically, unless there are associated clinical features of an underlying connective tissue disorder, such as Marfan’s syndrome.

Echocardiography is, therefore, an important investigation in PPS. Although assessment of the aortic valve and aortic root are key aspects of any echocardiographic examination, this is particularly pertinent in the young athlete. An important caveat to the use of echocardiography is that the aortic root is usually symmetrical in orthogonal planes however in certain aortopathies there can be asymmetric dilation, and so the typical PLAX view may underestimate the size of the aortic root. 3D echocardiography may be helpful in these athletes to better quantify the size of the aorta.

An aortic root dimension of > 39 mm (males) and > 33 mm (females) or scaled/height of > 20 mm/m (males) and > 19 mm/m (females) is likely to represent pathological dilation [56–58]. A universal sex-based cutoff however is fraught with danger as it may lead to smaller athletes with a comparatively large aortic root being passed as normal. It is important to scale the aortic root measurement in accordance with body size and, although body surface area has been used previously, the use of height alone removes inconsistencies related to weight change [56]. Similarly, it is essential to use a reproducible measurement technique for serial root measurements and the inner-edge to inner-edge methodology in end-diastole, defined as the onset of the QRS complex is recommended [71]. Care must be taken in interpreting the clinical significance of measurements in syndromic aortopathies. As we learn more about genotype/phenotype interactions the aortic size at which dissection occurs may be lower in certain high-risk genotypes [151].

### Mitral valve prolapse

Mitral valve prolapse (MVP) is defined as the systolic displacement of one or both mitral leaflets above the plane of the annulus and affects 1–3% of the general population [133]. Although it is a common cause of mitral regurgitation (MR) it is also associated with ventricular arrhythmia and SCD in young individuals [arrhythmic mitral valve prolapse (AMVP)]. The incidence of SCD secondary to AMVP is uncertain although it has been reported to account for 2–4% of SCD in competitive and non-competitive athletes [7]. Echocardiography can play an important role in patients with MVP by providing findings that may raise suspicion of an increased arrhythmic risk.

Arrhythmic MVP is typically associated with myxomatous degeneration and prolapse of both leaflets with a degree of mitral annular disjunction (MAD). Mitral annular disjunction describes the distinct separation between the MV annulus, the LA wall and the basal region of the LV myocardium [152]. While there is some guidance for the echocardiographic measurement of MAD length [153], with > 8.5 mm reportedly identifying 67% of patients who experienced non-sustained ventricular tachycardia on Holter [154], care must be taken to ensure accurate measurement when the posterior leaflet hinge-point becomes masked as the leaflet base becomes ‘muralised’ with the LA wall in late systole.

Lateral TDI may reflect abnormal myocardial mechanics secondary to bileaflet prolapse and hence can aid in identifying arrhythmic risk [155]. Lateral S’ that peaks in late systole (‘Pickelhaube sign’) and at a velocity > 16 cm/s has been proposed as a marker of greater arrhythmic risk associated with fibrosis and late Gadolinium enhancement on cMRI [155]. In those with proven ventricular arrhythmia, a high ectopic burden can lead to LV dilatation and impairment. In the setting of high ectopic burden, careful assessment of MR severity, including the duration of MR and its impact on regurgitant volume, is crucial to elucidate the true cause of LV dilatation and impairment.

Athletic cardiac remodelling is characterised by enlargement of all cardiac chamber dimensions and therefore, a mild degree of functional MR is common in athletes secondary to both LA and LV dilation. This, however, does not become haemodynamically significant when valve morphology is normal. The diagnostic challenge arises in athletes who present with MVP and moderate to severe chronic mitral regurgitation and LV dilatation. Consideration of interval changes in LV size or function are key. An increase in LV end-diastolic or end-systolic dimensions/volumes, or reduction in LVEF within a short time-frame and without a change in training type or volume are more likely to represent the adverse remodelling effects of chronic significant MR, especially when the LV diameter exceeds 60 mm [156]. Additionally, low forward resting SV is contradictory to the athlete’s heart and will reflect the large regurgitant volume. Estimating the regurgitant fraction in conjunction with SV is helpful in this setting with a low SV and a high regurgitant fraction indicative of pathological LV dilation secondary to significant MR.

The presence of severe MR will further elevate LV preload and reduce afterload and hence LV EF and GLS are often elevated in this setting, typically with values of greater than 60% and − 18%. Therefore, a finding of low LVEF or low GLS in an athlete with chronic severe MR should also raise the suspicion of impaired LV systolic function.

Left atrial dilatation is common in both athletic adaptation and MR. However, increases in LA volume secondary to athletic adaptation are likely to be accompanied by dilatation of all cardiac chambers, including the right atrium. Disproportionate dilatation of the LA is more likely related to the degree of MR in athletes with MVP. Similarly, an elevated resting pulmonary pressure should raise the suspicion of an increased haemodynamic burden from the co-existing MR and MVP.

## Additional assessment

### Exercise stress echocardiography

Acute aerobic exercise poses a significant challenge to the cardiovascular system. The heart responds with an elevation in HR and enhanced myocardial contractility/relaxation to provide a higher SV and cardiac output. Athletic conditioning results in physiological cardiac chamber enlargement and therefore the athlete is well conditioned in this regard to generate the higher cardiac outputs required for performance. Despite showing excellent function and HR during intense exercise [157], athletes often exhibit a lower resting LV and RV function (reduced EF, RVFAC and GLS) when compared to non-athletic individuals [20, 26, 46, 49].

The difference between resting and maximum contractility and HR is termed the cardiac reserve and secondary to lower basal values, this reserve is greater in athletes compared to the non-athlete [110], contributing to an enhanced cardiovascular performance. During exercise, there is also an increased oxygen demand imposed on the working myocardium. A healthy athletic individual with normal coronary arteries will have an enhanced supply of blood to match the increasing demand for oxygen and, hence, will be able to meet the required cardiac reserve.

Athletic individuals with inherited cardiomyopathy, myocarditis or underlying coronary artery disease have compromised myocardial function that may limit the heart in responding to the increasing challenges of exercise [20, 158–160]. This can be observed as a reduced cardiac reserve from a blunted functional response and/or direct impairment [50] and serves as the rationale for incorporating ESE in the routine evaluation of athletes with borderline resting ventricular function. The primary aim of ESE is to unmask the resting grey zone of the athlete heart and enhance the accuracy of diagnosing underlying cardiac disease.

Although there is anecdotal evidence to support bedside methods such as leg raises or squats, there is currently no agreed consensus on the specific protocols that should be employed for undertaking ESE in an athletic population [161]. It is however sensible to suggest that an athletic individual should undergo an exercise protocol (as opposed to a pharmacological stimulus) using a dedicated supine cycle ergometer where available. This allows for a true physiological stress whilst enabling diagnostic quality echocardiographic images. If a supine cycle ergometer is unavailable, then an upright cycle or treadmill can be used but are associated with the additional challenges to image quality when acquired during exercise. The design of the protocol should be aimed at pushing the athlete to a maximum HR (based on a value obtained from previous cardiopulmonary exercise testing or using the generally accepted equation of (220-athlete age) [162]). The maximum HR is achieved by gradually increasing the intensity of exercise through increments in workload with the magnitude of the increments being flexible and dependent on the athlete’s cardio-respiratory fitness. For example, an increase of 50 watts every 2 min would allow a very fit athlete to reach their maximum HR within 10 min whereas increments of 30 watts would allow a similar test duration in a less fit individual. The protocol should always include a recovery period allowing the HR and contractile state to return to close to baseline levels. Echocardiographic images should be acquired at baseline, throughout the protocol at each workload increment and into recovery. An alternative is to acquire images at a percentage of the athlete’s HR reserve (defined as *maximum HR—basal HR* [162]) which allows for direct comparison between individuals of varying fitness.

**Fig. 8:**
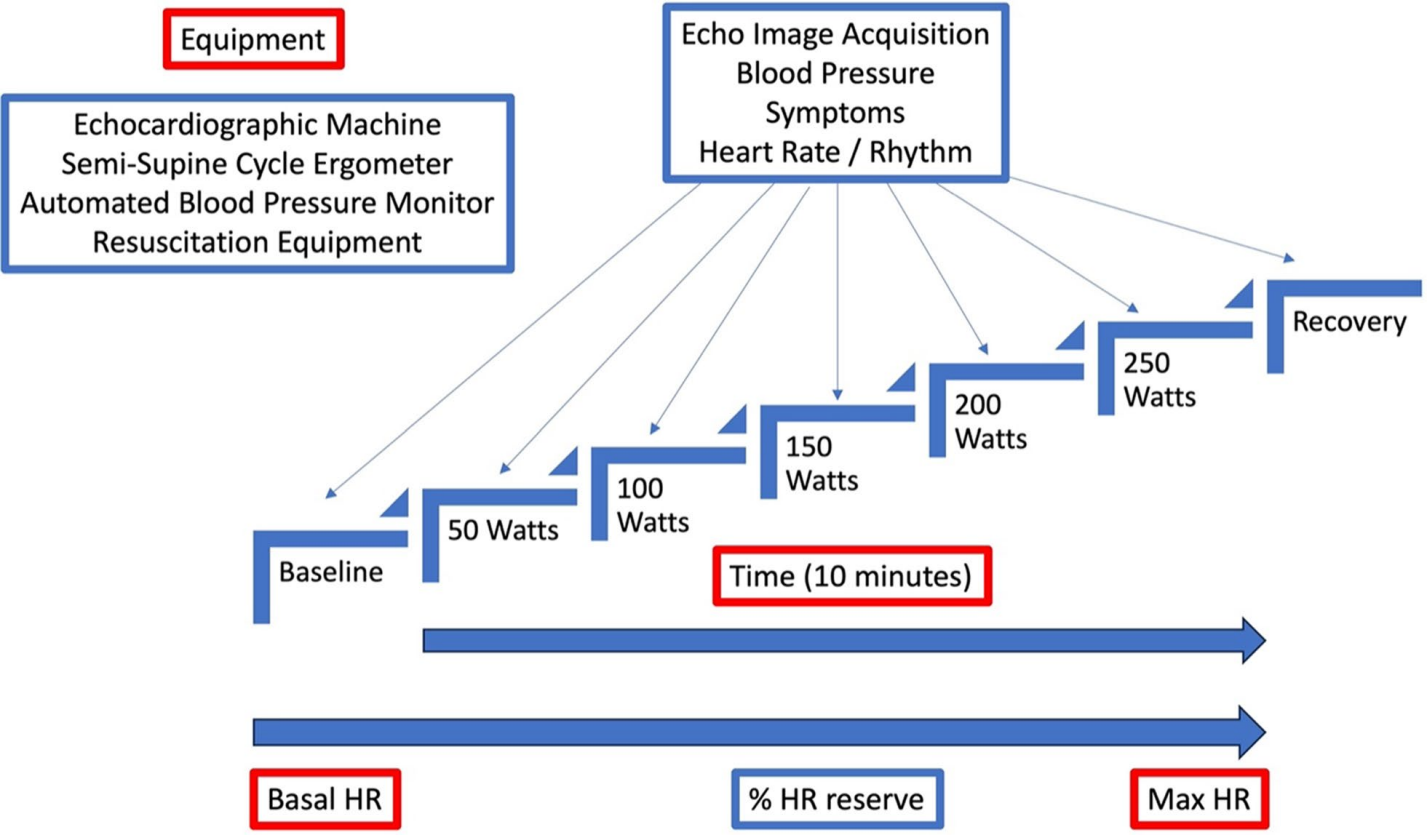
Suggested exercise echocardiography protocol

The choice and prioritisation of echocardiographic images should be focused and relative to the differential disease for assessment but should address the balance between clinical value and time. Alongside acquiring the echocardiographic images (with inherent rhythm ECG), systemic blood pressure, HR and symptoms should also be recorded. Figure 8 provides an exemplar protocol for ESE in an athletic individual.

### 3D echocardiography

Three-dimensional echocardiography enables comprehensive assessment of cardiac volume, providing a more accurate view of the morphological changes in the athlete’s heart compared to 2D imaging. While data on 3DE assessment of the athlete’s heart are limited, current 3DE studies consistently reveal ventricular enlargement and lower resting function in athletes with high endurance training, mirroring those obtained by 2D echocardiography [21, 163, 164]. Noteably, 3DE LV volume assessments demonstrate greater concordance with cMRI [165] than conventional echocardiography in various non-athlete populations and in a relatively small but well characterised athlete population [163]. Importantly this study highlighted the capacity to detect changes over time with analogous findings observed between modalities at a 2-year follow-up. These findings were reciprocated in RV volumes and EF, which highlights arguably the greatest benefit of 3DE. The non-uniform RV geometry constrains standard 2D assessments, particularly in quantifying the combined volume and function of both the inflow and the outflow. This limitation is critical when evaluating regional dysfunction in certain cardiomyopathies such as ACM. Data from cMRI highlights unique RV remodelling in endurance athletes and its correlation with exercise capacity [166], particularly dilatation towards the outflow of the infundibulum. This data alongside evidence of distinct 3D mechanics [21] underscores the potential added value of 3DE in this setting. We, therefore, acknowledge the role of 3DE as a follow-up investigation where conventional imaging is inconclusive or identifies potential maladaptation and regional dysfunction. The diagnostic algorithms presented here highlight the role of 3DE as a second line investigation.

## Conclusion

Echocardiography is a fundamental tool in the assessment of the young athlete’s heart. These guidelines highlight the importance and the effective role of echocardiography in the differentiation of physiological from pathological adaptation. The systematic approach is detailed within and includes the integration of pre-echocardiographic Information, the standardised examination, and the evidence-based interpretation along with the potential for an extended role of additional modalities including ESE and 3DE. With adherence to these guidelines, the BSE and Cardiac Risk in the Young, aim for improved detection of cardiovascular disease in this population and highlight this as ‘best practice’ in the assessment of the young athlete.

## Data Availability

No datasets were generated or analysed during the current study.
